# Investigating the metabolite signature of an altered oral microbiota as a discriminant factor for multiple sclerosis: a pilot study

**DOI:** 10.1038/s41598-024-57949-4

**Published:** 2024-04-02

**Authors:** Léo Boussamet, Emmanuel Montassier, Camille Mathé, Alexandra Garcia, Jérémy Morille, Sita Shah, Emilie Dugast, Sandrine Wiertlewski, Mathilde Gourdel, Corinna Bang, Klarissa H. Stürner, Damien Masson, Arnaud B. Nicot, Nicolas Vince, David-Axel Laplaud, Douglas L. Feinstein, Laureline Berthelot

**Affiliations:** 1 Nantes Université, Inserm, CHU de Nantes, CR2TI (Center for Research On Transplantation and Translational Immunology), 30 Bd Jean Monnet, 44000 Nantes, France; 2 Emergency Department, Nantes Hospital, Nantes, France; 3 Neurology Department, Nantes Hospital, Nantes, France; 4 Mass Spectrometry Platform, SFR Bonamy, Nantes, France; 5 Institute of Clinical Molecular Biology, Christian Albrechts University of Kiel, Kiel, Germany; 6 Department of Neurology, University Hospital Schleswig-Holstein, Kiel, Germany; 7 Clinical Biochemistry Department, Nantes Hospital, Nantes, France; 8 Jesse Brown VA Medical Center, 835 South Wolcott Ave, MC513, E720, Chicago, IL 60612 USA; 9 Department of Anesthesiology, University of Illinois, Chicago, IL USA

**Keywords:** Multiple sclerosis, Saliva, Gut, Microbiota, Metabolites, Immunology, Microbiology, Pathogenesis

## Abstract

In multiple sclerosis (MS), alterations of the gut microbiota lead to inflammation. However, the role of other microbiomes in the body in MS has not been fully elucidated. In a pilot case-controlled study, we carried out simultaneous characterization of faecal and oral microbiota and conducted an in-depth analysis of bacterial alterations associated with MS. Using 16S rRNA sequencing and metabolic inference tools, we compared the oral/faecal microbiota and bacterial metabolism pathways in French MS patients (n = 14) and healthy volunteers (HV, n = 21). A classification model based on metabolite flux balance was established and validated in an independent German cohort (MS n = 12, HV n = 38). Our analysis revealed decreases in diversity indices and oral/faecal compartmentalization, the depletion of commensal bacteria (*Aggregatibacter* and *Streptococcus* in saliva and *Coprobacter* and *Roseburia* in faeces) and enrichment of inflammation-associated bacteria in MS patients (*Leptotrichia* and *Fusobacterium* in saliva and Enterobacteriaceae and *Actinomyces* in faeces). Several microbial pathways were also altered (the polyamine pathway and remodelling of bacterial surface antigens and energetic metabolism) while flux balance analysis revealed associated alterations in metabolite production in MS (nitrogen and nucleoside). Based on this analysis, we identified a specific oral metabolite signature in MS patients, that could discriminate MS patients from HV and rheumatoid arthritis patients. This signature allowed us to create and validate a discrimination model on an independent cohort, which reached a specificity of 92%. Overall, the oral and faecal microbiomes were altered in MS patients. This pilot study highlights the need to study the oral microbiota and oral health implications in patients with autoimmune diseases on a larger scale and suggests that knowledge of the salivary microbiome could help guide the identification of new pathogenic mechanisms associated with the microbiota in MS patients.

## Introduction

With more than 10 trillion bacteria populating the human skin, gut and airway, the microbiome harbours a symbiotic relationship with its human host. A very precise equilibrium is maintained between the microbiota and the host immune system, which must tolerate commensal flora while initiating appropriate inflammatory responses towards potential pathogens^1^. To maintain this balance, evolution favoured members of human the microbiota with immune-stimulating traits, often referred to as proinflammatory bacteria, as well as bacteria with regulatory properties, which are known as regulatory bacteria. Long-term perturbations of this balance can lead to a pathological state^2^. In this context, dysbiosis of the microbiome has been associated with many inflammatory diseases. As a major part of the microbiota resides within the gut, initial reports examined diseases affecting the gut such as inflammatory bowel disease^3,4^, and coeliac disease^5,6^. Inflammatory diseases affecting other organs including the skin, such as acne^7^, atopic dermatitis^8^ and psoriasis^9^, have also been shown to be associated with both skin and gut microbiome. In addition to the gut, oral dysbiosis has been observed in a variety of conditions, including oral periodontitis^10^ as well as systemic diseases such as systemic erythematosus lupus^11^ and IgA vasculitis^12^ and in organ specific diseases like rheumatoid arthritis (RA)^13^ and Alzheimer disease^14^, where *Porphyromonas gingivalis* seems to be actively involved. More recently, other inflammatory diseases such as multiple sclerosis (MS), an inflammatory disease of the central nervous system, have exhibited changes in microbiota density or composition.

**Figure 1:**
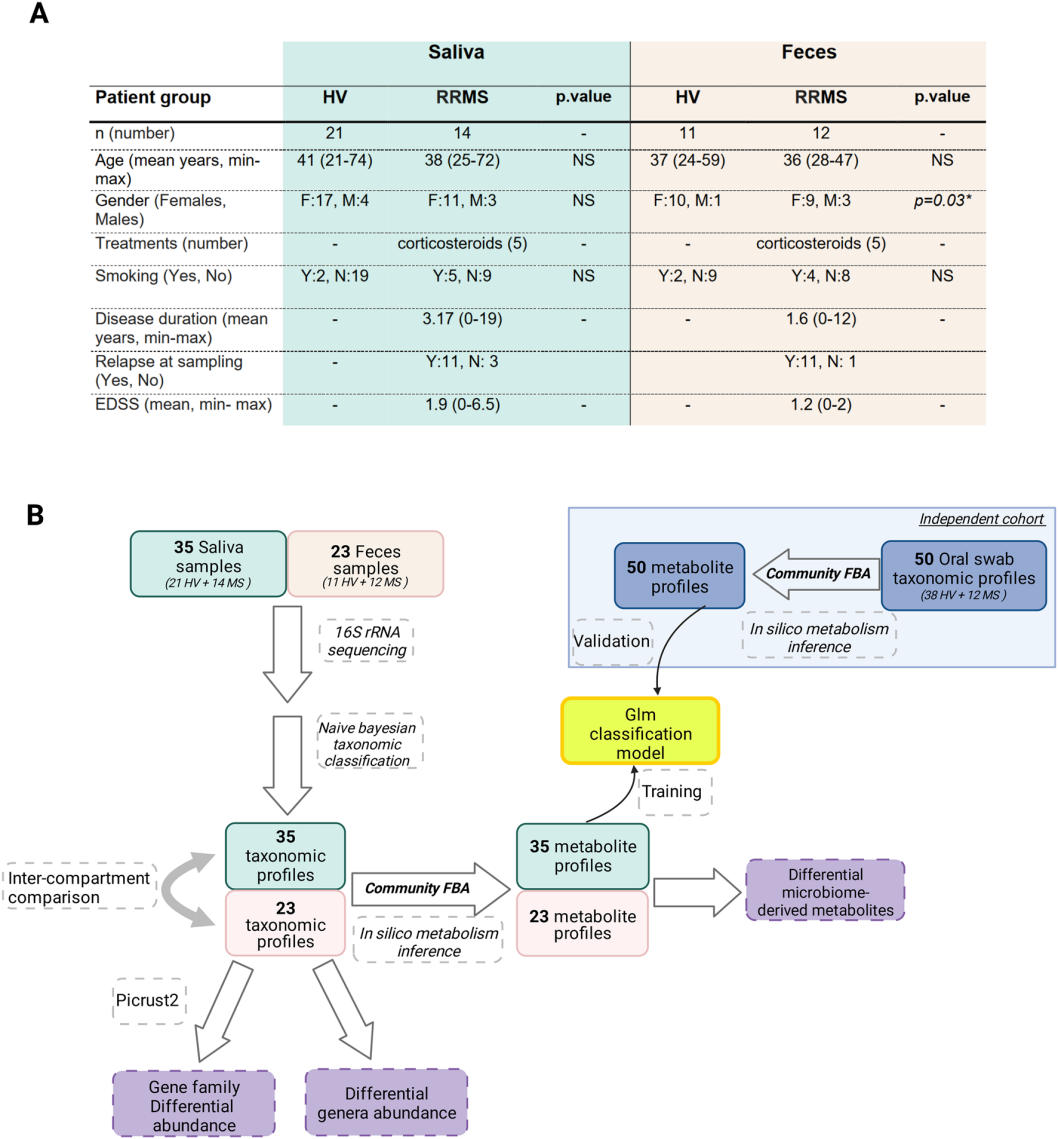
Patient cohort and sample processing. (**A**) Patient clinical characteristics. (**B**) Included faeces and saliva samples were investigated regarding their bacterial and microbiome-derived metabolites composition. Taxonomy was obtained based on 16S rRNA sequencing and Naive Bayes classification algorithm. Microbiome-derived metabolites were inferred by community Flux Balance Analysis (FBA) and metagenomic gene content was inferred using the PICRUSt2 algorithm^28^. Oral metabolites profiles were used to construct a classification model that was further validated using an independent cohort.

The faecal microbiota has been extensively studied in MS patients and exhibits traits that can be interpreted as moderate dysbiosis^15–17^. Several animal studies have shown that the gut microbiome can actively participate in disease physiopathology. Indeed, studies on experimental autoimmune encephalomyelitis (EAE), a murine model of MS, have revealed that gut microbiota plays a role in demyelinating diseases. Indeed, disease induction in germ-free mice resulted in milder symptoms^18^, associated with decreased proinflammatory cytokine production^19^. Consistent with this, faecal transplantation from MS patients to germ-free mice (genetically modified to spontaneously develop EAE)^20^ resulted in a dramatic increase in spontaneous EAE incidence; this result was reproduced using the classical MOG_35-55_ peptide-induced EAE model^21^. However, while the faecal microbiome has been extensively studied, whether the oral microbiome is impacted by or contributes to MS, including both the microbial composition and functional aspects, has not been determined. Indeed, very few studies have considered these factors^22–24^. Preliminary works have reported the presence of particular bacteria or fungi in the oral and nasal mucosa, including a trend towards a higher frequency of *Candida spp*^25^ and *Staphylococcus aureus* virulence markers^26,27^ in MS. Overall, the fact that the oral microbiota has already been associated with several other inflammatory diseases and the proximity of the latter to the tonsils, the dental nerves, and the deep cervical lymph nodes makes it an interesting candidate in the context of a CNS inflammatory disease mediated by peripherally educated immune cells. In this pilot study we explored broad aspects of the microbiota and were able to obtain initial answers to these questions. In the present study, we investigated the changes in the microbiota of relapsing–remitting (RR) MS patients in both the oral and gut compartments compared to healthy volunteers (HVs) (Fig. 1). Our aim was to study the oral and faecal microbiota both separately and jointly and we treated them as interconnected compartments. On the basis of 16S rRNA sequencing results, we reported that both the oral and faecal microbial communities, as well as their interplay, are altered in MS. While alterations found in faecal microbiota are in line with the current literature, oral microbiota alterations appear to be more pronounced, as suggested by the diversity analyses. We further identified a specific signature in oral microbiota-derived metabolite and altered microbial gene expressions. We used these data to generate a discrimination model, which we then validated in an independent cohort.

## Results

### The abundance of bacterial genera in the in salivary and gut microbiota from MS patients is altered

**Figure 2:**
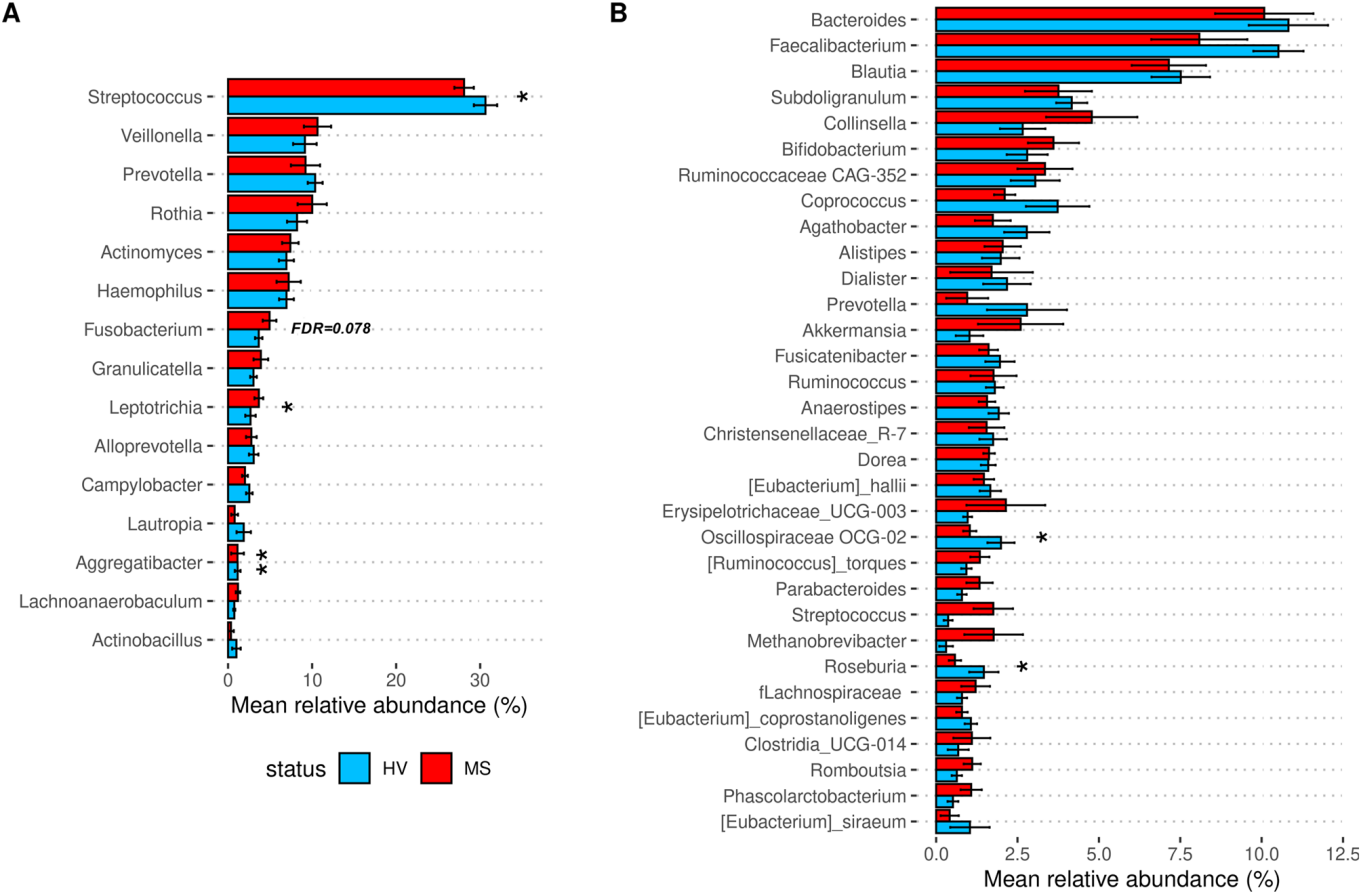
Relative abundance of most abundant bacterial genera. Taxonomic dataset was used to quantify the relative abundance of bacterial genera present in (**A**) oral and (**B**) faecal samples. Data are mean values obtained for genera in RRMS (red squares) and HV (blue squares) samples that were present at 1% RA or greater. *FDR < 0.05, **FDR < 0.01 from Wilcoxon rank-based tests. Error bars are represented by standard error (SEM).

As summarized in the workflow in Fig. 1B, we first compared the relative abundance of bacterial genera found in RRMS to that in HV samples from individuals. Basic quality controls performed on the sequencing products revealed high-quality reads and a sequencing depth above 10K dereplicated reads for all samples. In addition, blank sequencing controls revealed an absence of bacterial contamination. Visual inspection via alpha rarefaction analysis revealed that all the samples had reached a diversity plateau, suggesting that the sampling effort was sufficient to capture most of the microbial diversity present in our dataset.﻿ We identified 108 individual genera in the salivary samples and 236 in the faecal samples (Tables S1 and S2). In the saliva of RRMS patients (Fig. 2A), 13 genera were present at a mean relative abundance > 1%, and these genera made up almost 92% of the entire microbiota. In HV saliva, 14 genera were also present at a 1% relative abundance or greater, and these genera made up 89% of the total microbiota. Significant reductions in relative abundance (Table S1) of two of these bacteria (*Streptococcus*, *Aggregatibacter*) were observed between the RRMS and HV cohorts, and two bacteria (*Fusobacterium, Leptotrichia*) had significantly increased abundance or tended to be more present in the RRMS cohort (FDR = 0.08 and FDR = 0.04, respectively). Furthermore, the relative abundances of three genera (*Catonella, Dialister, Paludibacteraceae-F0058*) were significantly lower in the RRMS group than in the HV group while that of four others were increased (*Mobiluncus*, *Cryptobacterium*, *Howardella* and *Lactobacillus*). In addition, linear discriminant analysis (LDA) revealed that *Cryptobacterium* was associated with RRMS while *Streptococcus* and unclassified members of the families Burkholderiaceae and Pasteurellaceae were associated with HV (Fig. S1A).

Examination of the gut microbiota (Fig. 2B) revealed no modification of general diversity (Fig. S2), but 28 genera were present in RRMS patients with a mean relative abundance > 1% and these genera accounted for 74% of the total microbiota. In the HV group, 24 genera had a relative abundance > 1.0% and they comprised 73% of the total microbiota. A comparison of the mean relative abundance values revealed that *Oscillospiraceae UCG-002* and *Roseburia* abundances were significantly lower in the RRMS group than in the HV groups (Table S2). There were 20 genera with lower abundances in RRMS samples than in HV samples (*Intestinibacter, Escherichia-Shigella, Ruminococcaceae-UBA1819, Varibaculum, Actinomyces, Eisenbergiella, Hungatella, Corynebacterium, Gemella, Peptoniphilus, Rothia,* Puniceicoccaceae member, *Fusobacterium, Paludicola, Solobacterium, Acetanaerobacterium, Papillibacter, Clostridium, Peptostreptococcus* and Lachnospiraceae-GCA-900066755), and the abundances of ten genera were decreased (*Oscillospiraceae_UCG-002*, *Roseburia*, two *Eubacterium* members, *Lachnospiraceae_UCG-001*, *Coprobacter*, *Staphylococcus* and three Prevotellaceae members). These results largely overlap with the results of the LDA analysis of the gut microbiota (Fig. S1B). Moreover, the abundance of *Akkermansia* tended to increase in patients with RRMS (mean 1% in HV versus 2.6% in RRMS). Overall, no bacterial genera existing in either the oral and faecal samples were altered, although the relative abundance of *Fusobacterium* tended to increase in both compartments (false discovery rate (FDR) = 0.08 in saliva and FDR < 0.05 in faeces).

### A dysbiotic state and decreased microbial oral diversity were observed in RRMS patients

**Figure 3:**
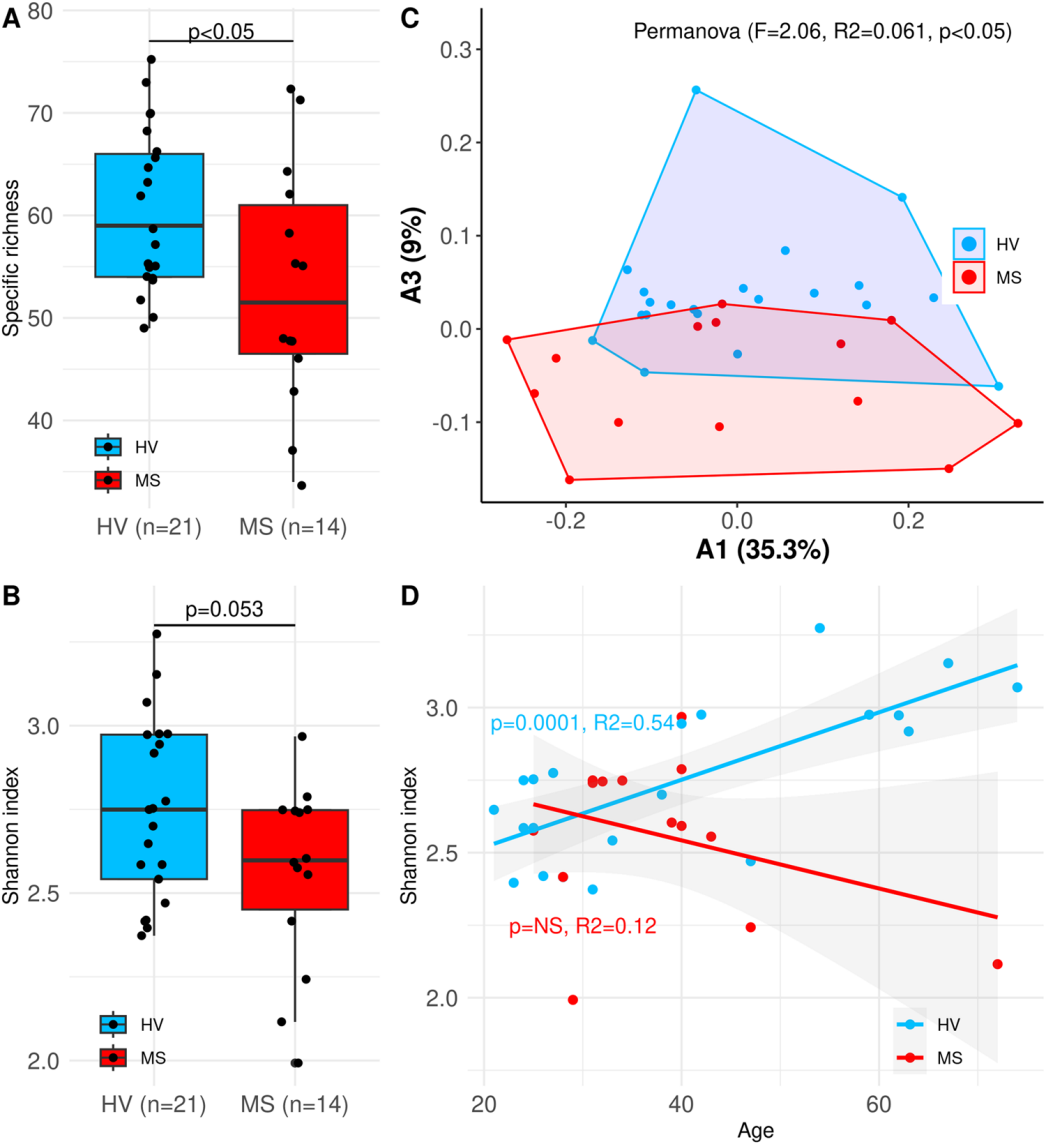
Global oral microbiome alterations in RRMS. (**A**) Shannon index and (**B**) Specific richness are decreased in RRMS patients (Wilcoxon tests). (**C**) Principal coordinate analysis (PCoA) individual projection. The PERMANOVA test showed signs of oral dysbiosis in RRMS (p < 0.05). (**D**) The oral Shannon diversity increases with age in HV but not in MS patients.

A comparison of the oral bacterial diversity between our RRMS cohort and the HV cohort revealed a trend towards a decrease in specific richness (Fig. 3A) and a significant decrease in the Shannon index (Fig. 3B) compared to that of the HV cohort. Additionally, β-diversity analysis by permutational multivariate analysis of variance (PERMANOVA, Fig. 3C) indicated that the composition of the oral microbiota of RRMS patients differed from that of the control group. Interestingly, we observed that the Shannon index significantly increased with age in HVs, but not in RRMS patients (Fig. 3D). Taken together, these data reveal the dysbiotic state of the oral microbiota in patients with RRMS. We also detected signs of faecal dysbiosis (PERMANOVA, p < 0.05) but there was no significant association between the α-diversity indices and disease status (Fig. S2).

### There is more similarity between the oral and faecal compartments in MS patients than HV

**Figure 4:**
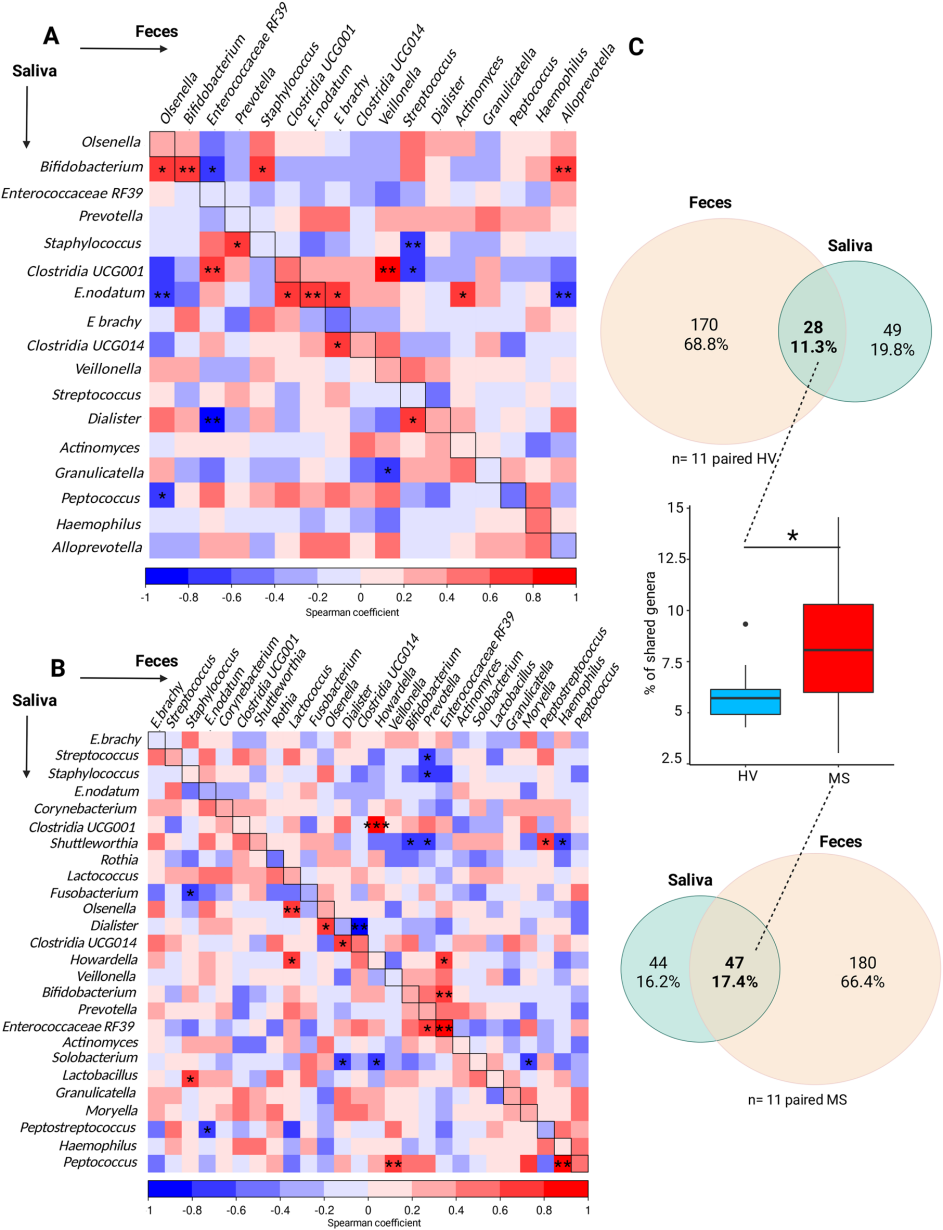
Comparison of oral and faecal microbiome compositions. Spearman correlation coefficients were determined comparing the relative abundance of the oral versus faecal bacterial genera in the (**A**) HV and (**B**) RRMS samples. Red colour represents a positive association; blue represents a negative association. The horizontal axis represents the faecal composition and the vertical axis represents the oral composition. Statistically significant spearman correlations are indicated by stars: *p < 0.05, **p < 0.01. (**C**) The numbers of shared genera between oral and faecal flora in HV (upper Venn diagram) and in MS (lower Venn diagram). The middle boxplot represents the number of shared genera per individual.

Using paired faecal and saliva samples from 11 RRMS patients and 11 HV, we identified specific correlations between the two compartments in the RRMS and HV groups. In the healthy group (Fig. 4A), 21 significant correlations were found between the oral and faecal compartments, which is consistent with good compartmentalization; 13 of these correlations were positive (Fig. 4A, red squares) and 8 were negative (Fig. 4A, blue squares). Interestingly, *Eubacterium nodatum* and *Bifidobacterium* were positively correlated with each other in the oral and faecal compartments. The other bacteria that were strongly correlated between both compartments included oral *Bifidobacterium* and faecal *Alloprevotella.* Negative correlations were found between oral *Eubacterium nodatum* and faecal *Alloprevotella,* oral *Dialister* and faecal Enterococcaceae RF39 and oral *Staphylococcus* and faecal *Streptococcus*. Intriguingly, all these correlations were absent in the RRMS group and new correlations appeared, suggesting a different dynamic within the oral and intestinal interplay (Fig. 4B). Indeed, in the RRMS group, 24 significant correlations were detected between the gut and saliva, 13 of which were positive correlations; of these correlations the most significant corresponded to oral and faecal *Enterococcaceae RF39*, two different clostridia species, oral *Peptococcus* with faecal *Haemophilus* and *Veillonella*. The remaining 11 correlations were negative correlations mainly including oral *Dialister* and faecal Clostridia UCG014. When all the individual samples from the HV and RRMS groups were pooled (Fig. 4C), 28 (11.3%) genera were shared between the two compartments in the HV group whereas 47 (17.4%) were shared in the RRMS group. This difference was confirmed at the individual level by comparing the mean percentage of shared genera in each group via the Wilcoxon test (p < 0.05). As both the proportions of oral-specific and faecal-specific genera decreased in RRMS patients, it seems that faecal residents can colonize the oral cavity and vice versa. Taken together, these results suggest that compartmentalization tends to breakdown in MS.

### Alterations in bacterial genes in MS

**Figure 5:**
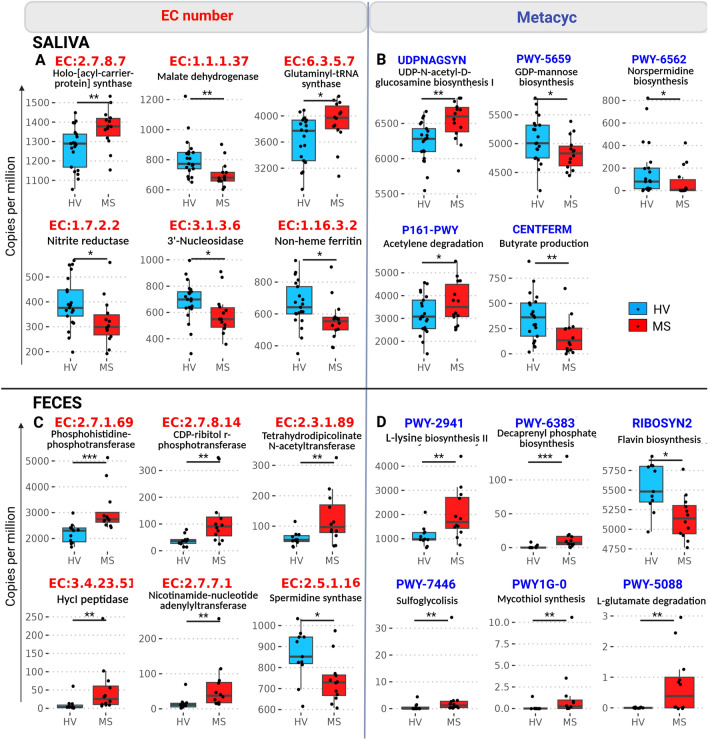
Functional metagenomic profiling. PICRUSt2 algorithm was used to infer gene family abundances, presented in count per million reads and group by EC numbers [(**A**) for Saliva and (**C**) for Faeces] and by Metacyc pathways [(**B**) for Saliva and (**D**) for faeces]. The most differentially abundant gene families between HV and RRMS groups are shown. *FDR < 0.05, **FDR < 0.01, ***FDR < 0.001.

As directly measuring the gene content using 16S rRNA sequence data is not possible, we used the Phylogenetic Investigation of Communities by Reconstruction of Unobserved States (PICRUSt2^28^) algorithm to perform functional enrichment analysis by inferring gene content based on the microbiota data. The obtained gene copy numbers were then grouped into conserved families that share common biological functions such as enzymes (identified by EC numbers) or by bacterial Metacyc pathways (https://metacyc.org/). In the oral compartment, we identified six highly significant enzymatic pathways (Fig. 5A) and five metabolic pathways (Fig. 5B) that differed significantly between the RRMS and HV samples. We found a decrease in the malate dehydrogenase (EC1.1.1.37) level in patients with RRMS; malate dehydrogenase is a key enzyme of the Krebs cycle. In contrast, we detected an increase in holoacyl carrier protein synthase (EC:2.7.8.7), which is involved in fatty acid biosynthesis pathways. We also found that the acetylene degradation pathway (Metacyc P161-PWY, which produces acetate, increased and that of CENTFERM, which produces butyrate, decreased, suggesting a shift from butyrate production in HVs to acetate production in RRMS patients. Other alterations in the saliva of RRMS patients included a decreased abundance of families involved in stress responses such as nitrite reductase (nrfA, EC:1.7.2.2) and 3′-nucleosidase (EC:3.1.3.6). Finally, the UDP-*N*-acetyl-d-glucosamine biosynthesis pathway, which is important for bacterial surface glycoprotein synthesis, was increased (Metacyc UDPNAGSYN) in RRMS while GDP-mannose biosynthesis (Metacyc PWY-5659) and norspermidine biosynthesis (Metacyc PWY-6562 were decreased.

Examination of faecal samples revealed six inferred enzymatic pathways (Fig. 5C) and six Metacyc pathways (Fig. 5D) that differed significantly between the HV and RRMS samples. Increases in HycI peptidase (EC:3.4.23.51), which is involved in hydrogenase-3 maturation and has pH resistance properties^29^; and CDP-ribitol r-phosphotransferase (EC:2.7.8.14), which catalyses the production of teichoic acid, a gram-positive cell membrane component, were observed. There was also detection of amino acid-related pathways including phosphohistidine-phosphotransferase (EC:2.7.1.69), l-lysine biosynthesis II (Metacyc PWY-2941), and l-glutamate degradation (Metacyc PWY-5088). Pathways involved in mycothiol synthesis, decaprenyl phosphate biosynthesis and sulfoglycolysis pathways (Metacyc PWY1G-0, PWY-6383, and PWY-7446, respectively) were also more abundant in RRMS. Spermidine synthase (speE, EC:2.5.1.16), which catalyzes polyamine spermidine production and flavin biosynthesis (Metacyc RIBOSYN2) were significantly less abundant in the gut compartment of RRMS patients.

To evaluate the robustness of the PICRUSt2 algorithm generated utilizing our data, we performed quantitative PCR to validate two different targets of interest, one in the oral compartment (nrfA, EC:1.7.2.2) and one in the faecal compartment (speE, EC:2.5.1.16). We showed that these two targets had decreased gene copy numbers in the RRMS group, as predicted by the PICRUSt2 algorithm (Fig. S3).

### Microbiota-derived metabolites inference can be utilized to discriminate MS patients from HVs and RA patients

**Figure 6:**
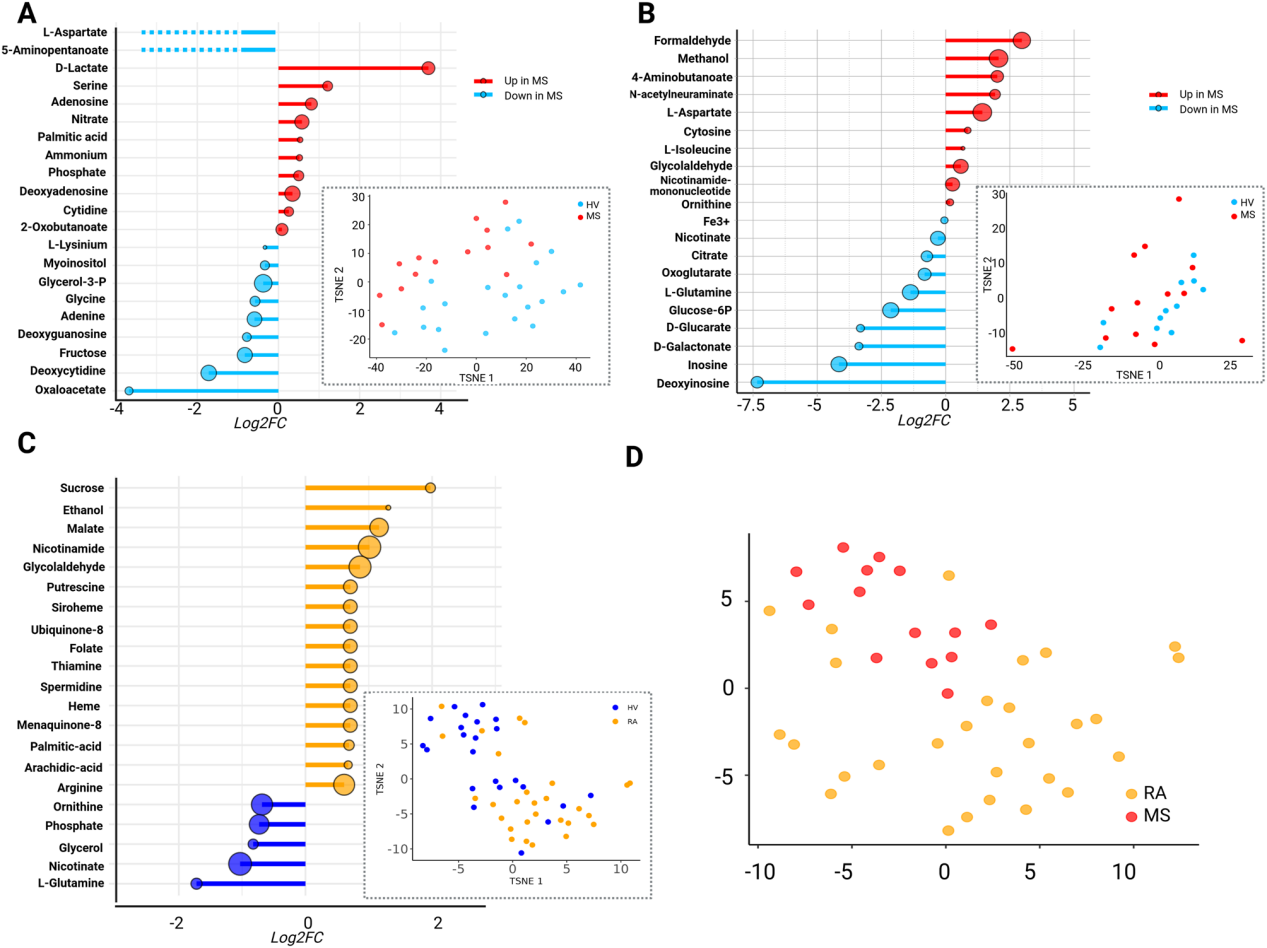
Predicted availability of microbiota-derived metabolites. Bacterial metabolism was reconstructed using FBA. For each individual 80–90% of their total microbiome was reconstituted. Circle size represents significance level. Dotted lines represent metabolites switches from production to consumption (blue dotted lines) (**A**,**B**) Significantly affected bioavailability of microbiota derived metabolites in MS and individual metabolite fluxes t-distributed stochastic neighbour embedding (t-SNE) projection in (**A**) Saliva, and (**B**) Faeces, expressed in log_2_ (Fold Change). (**C**) Significantly affected bioavailability of microbiota-derived metabolites in the saliva of RA and corresponding individual metabolite fluxes t-SNE projection. (**D**) Metabolism reconstruction for RA and MS were merged and the corresponding fluxes were projected on a t-SNE reduction. Results were considered significant using p < 0.05 and FDR < 0.1.

As microbiota-derived metabolites can have important effects on host responses, we used the flux balance analysis (FBA) algorithm to predict metabolite availability. Globally, 80 to greater than 90% of the total microbiota were reconstituted for each community model for each patient (Fig. S4). Metabolite bioavailability indices were inferred by converting boundary reactions, whose fluxes correspond to secreted or consumed metabolites according to the community model, into the fold-change of the reactional fluxes between HVs and RRMSs. In saliva (Fig. 6A), several metabolites, including d-lactate, ammonium, nitrate, palmitic acid, and nucleic acids including adenosine, deoxyadenosine, and cytidine were predicted to be over-produced in RRMS samples. In contrast, several metabolites including myoinositol, fructose, oxaloacetate, aminopentanoate and deoxycytidine exhibited decreased bioavailability. The LDA showed comparable results (Fig. S1C).

In the faecal microbiota (Fig. 6B), the predicted bioavailability of a distinct set of metabolites was affected. Formaldehyde and glycolaldehyde, methanol, 4-aminobutanoate, nicotinamide mononucleotide, the amino-acids l-aspartate and l-isoleucine, and the nucleoside cytosine showed increased bioavailability in RRMS samples. Decreased bioavailabilities of the nucleosides inosine and deoxyinosine; the Krebs cycle intermediates citrate and oxoglutarate, and the sugars d-glucarate, d-galactonate, and glucose-6-phosphate were observed, confirmed by LDA (Fig. S1 D).

**Figure 7:**
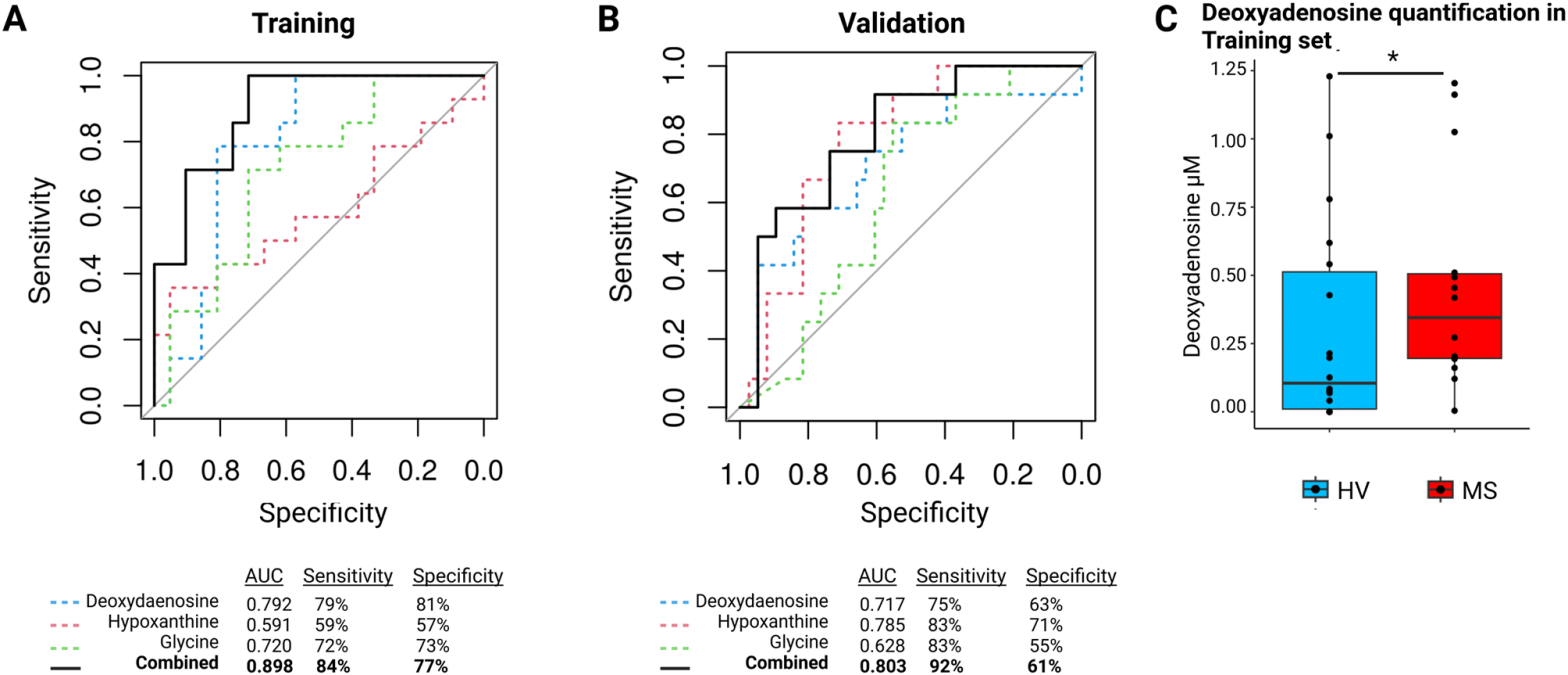
Discrimination of HV from MS groups using metabolic profiles. Receiver operator characteristic (ROC) curves for (**A**) the training dataset (saliva samples, 21 HV, 13 MS) and (**B**) validation dataset (swabs samples, 38 HV, 12 MS) based on microbiota-derived metabolites deoxyadenosine, hypoxanthine, and glycine. (**C**) Confirmation by direct mass spectrometry quantification of the top discriminating metabolite corresponding to deoxyadenosine in the oral cavity of RRMS (n = 13) compared to HV (n = 13) using mass spectrometry. Wilcoxon test: p < 0.05.

To determine whether microbiota-derived metabolites could be utilized to discriminate between controls and RRMS patients, we performed t-distributed stochastic neighbour embedding* (t-SNE)* variable reduction, and the projection arising from oral microbiota-derived metabolites (Fig. 6A, t-SNE plot) better discriminated disease status than arising from faecal microbiota-derived metabolites (Fig. 6B, t-SNE plot). Interestingly, the signature of the oral microbiota-derived metabolites of RRMS patients appeared to be distinct from that observed in rheumatoid arthritis (RA), as shown in Fig. 6C,D. Indeed, most oral metabolites whose levels are altered in RA (Fig. 6C) are different from those altered in MS and the bioavailability of only palmitic acid was increased in both diseases. Discrimination between MS and RA patients on the basis of oral metabolites is represented on the t-SNE reduction in Fig. 6D. We therefore decided to use oral microbiota-derived metabolites to develop a classification model based on logistic regression with automatic variable stepwise selection (*Akaike*'*s information criterion* minimization). The model identified deoxyadenosine (p = 0.009), hypoxanthine (p = 0.06) and glycine (p = 0.09) as the best classifiers according to our training dataset, giving an area under the receiver operating characteristic curve (AUC) of 0.91 (Fig. 7A). To validate this model, we used an independent German cohort^23^. We inferred the microbiota-derived metabolites of the 38 HVs and the 12 treatment-free RRMS patients. This achieved an AUC of 0.80 (Fig. 7B) consistent with the possibility that RRMS patients can be distinguished from HVs according to their oral metabolite content. To ascertain the predictive ability of our metabolite model, we measured the levels of the most discriminating metabolite, deoxyadenosine, in saliva samples by mass spectrometry. The level of this metabolite, which was predicted to increase in the RRMS group was indeed increased according to direct measurement (Fig. 7C), thus substantiating our FBA model.

## Discussion

In this study, we identified a dysbiotic state in both the oral and faecal microbiota of MS patients compared to HVs. Among the oral microbiota, five genera (*Dialister, Aggregatibacter, Catonella, Streptococcus, and Paludibacteriaceae-F0058*) had a decreased relative abundance in the MS cohort. Interestingly, the first three genera have also been shown to be associated with periodontal diseases^10^. In contrast, several proinflammatory bacteria and potential pathogens, such as *Fusobacterium* and *Leptotrichia* had an increased abundance in the MS oral microbiota. Despite a potential leverage effect due to enrichment in individuals older than 60 years in the control group as shown in Fig. 3, we observed an interesting age-dependent increase in Shannon diversity in the HV cohort but not in the MS cohort. Age-related increases in gut microbiome diversity have been reported^30,31^ and could reflect an increase in diet complexity and nutrients. Dysbiosis of the oral microbiome in MS patients has been previously described. In a longitudinal study of oral swabs^23^, there were significant increases in the relative abundance of *Campylobacter, Haemophilus *and* Neisseria*. In a second study^24^, DNA sequencing of cultured bacterial isolates from oral swabs by PCR revealed that *Staphylococcus, Actinomyces, Fusobacterium, Bacteroides, Porphyromonas, Prevotella, Veillonella* and *Propionibacterium* were significantly more abundant in the MS group than in the control group, while *Lactobacillus* and *Peptostreptococcus* were more prevalent in the HV group. Together these studies confirm that oral dysbiosis is a feature of MS similar to other neurodegenerative diseases. The contribution of the reported oral dysbiosis to the disease remains to be addressed. Even though our cohort of patients was diagnosed relatively early in the course of the disease and exhibited only mild symptoms, we cannot rule out the possibility that some of these alterations could be attributed to difficulties in maintaining oral health due to muscle and coordination issues, which are common symptoms of MS. Potential causality and direct contribution of the oral microbiome to the disease should be further evaluated experimentally and through Mendelian randomization analysis.

With respect to the gut microbiota, we found similar types of alterations oriented towards a proinflammatory environment. Indeed, the abundances of several of the major short-chain fatty acid (SFCA) producers were decreased (*Prevotella* and *Roseburia*). In addition, the abundance of *Fusobacterium* increased in the gut as it did in the saliva. Finally, the *Enterobacteriaceae* family (including *Escherichia*, *Shigella*, *Enterobacter* and others) exhibited a large increase in abundance in the MS group (up to 2.5% of their total microbiota, or a 450% increase in the MS group) while the abundance of this group was close to zero in the HV group. Our results regarding faecal microbiota are consistent with the current literature, including the recently published study in Cell by the iMSMS consortium^17^. That study, as several others, have shown a tendency towards a decreased abundance of *Faecalibacterium* and an increase of *Akkermansia* abundance in MS. Similarly, our findings of a decreased abundance of the genus *Roseburia* have been previously described^32^, as were the increases in the abundance of *Actinomyces*^33^, *Hungatella*^34^ and different genera of *Ruminococcus*^34^.

We compared the microbiota compositions in the oral and faecal samples and found a high degree of compartmentalization with only a quarter of all the reported genera capable of living in both regions, consistent with microbiomes having location-specific compositions. However, there were more shared genera in MS patients than in HVs, suggesting a loss of compartmentalization during MS. Whether the exchange between the two compartments is bidirectional, or how transmission occurs (circulation, digestion, faecal–oral route) remains to be determined.

Among the metabolites, oral ammonium, which is a potent neurotoxic compound, was more common in the oral cavity of MS patients. On the other hand, myoinositol and deoxycytidine showed decreased bioavailability in MS patients. Myoinositol is an important cell messenger and osmolyte in the nervous system. Deoxycytidine is a hypomethylating agent, that has promising protective effects and is capable of improving the course of EAE^35^. Analysis of bacterial gene family content also revealed alterations in microbiota pathways. Indeed, the activity of malate dehydrogenase, which is a limiting factor in the TCA cycle, was decreased. Compensation seemed to occur by increasing lactic acid fermentation. Moreover, although the TCA cycle slowed, citrate did not accumulate but instead may have been used for fatty acid biosynthesis as suggested by the increase in the abundance of holo-acyl-carrier synthase.

The reorganization of bacterial species also affects bacterial pathways and metabolite availability in the gut. Indeed, our models predicted a large increase in the production of methanol and formaldehyde, and a decreased availability of inosine and deoxyinosine. Although best known for its cancerogenic properties, formaldehyde was also reported to play a role in neurological disorders^36^. Conversely, inosine was shown to improve cognitive function and to decrease neuroinflammation in ageing rats^37^. Strikingly, HycI peptidase, an enzyme from *Escherichia coli* that acts as a pH resistance factor (pH 2–2.5)^29^, was more abundant in MS. As the acidity of the stomach represents a major barrier between the oral and faecal compartments, increased activity of this enzyme play a role in the decompartmentalization between the gut and oral cavity observed in MS. Interestingly, the abundance of Actinomyces genus, along with several *Actinomycetales* compounds increased in the gut of MS patients. Notably, there is a peptide from *Actinomyces* can mimic collagen type IV α3_127–148_ and become a source of autoimmunity, thus leading to anti-glomerular basement membrane disease in HLA DRB1*15:01 humanized mice^38^. A significant decrease in spermidine synthase by the gut microbiota, as well as a decrease in components of the norspermidine biosynthesis pathway in the saliva was observed. Spermidine is a polyamide involved in cell proliferation, and has regulatory properties^39^.

Finally, our analysis suggested that the predicted levels of microbiota-derived metabolites can be used to discriminate between MS and HV. This result was validated using an independent cohort of swab samples. Despite differences in bacterial abundances between swabs and saliva samples, as observed by Kaan et al.^40^, the corresponding metabolites produced appeared convergent. The discriminant metabolites can be used as a surrogate biomarker for monitoring the efficacy of therapeutic intervention. Moreover, the MS metabolite signature is distinct from that of RA. This indicates a specific MS oral signature and not just an inflammatory signature in a wider sense. However, whether these alterations are causative or a consequence of the disease remains unknown. Several ongoing clinical trials that include modulation of the microbiome should help answer these questions. Moreover, several studies suggest that periodontal pathogens can trigger neuroinflammation^41^. In an EAE model, the induction of experimental periodontitis exacerbated the symptoms of neuroinflammation and faecal microbiota transplantation from these mice also aggravated the disease^42^.

Taken together, our results confirm the microbiota alterations in both oral and faecal compartments in MS patients. We identified relevant bacteria and microbiota-derived metabolites, that could favor a proinflammatory microenvironment. This also highlights the fact that the oral health MS patients should be monitored. Moreover, we identified several microbial pathways shared with human metabolism in both the saliva and gut. Using a modest sample size and three explicative variables in our classification model, we were able to show that oral microbiome-derived metabolites can be utilized to discriminate between MS patients and HVs. Metagenomic and metabolomic studies will be very informative to validate the results of this pilot study on the oral microbiota.

## Conclusions

This pilot study confirmed the feasibility and relevance of studying not only the faecal microbiome but also the oral microbiome in multiple sclerosis patients. The present study offers compelling insights into the dynamics of the microbiota in MS, as well as several bacterial pathways possibly that could impact human immunology. In this detailed analysis, we identified relevant metabolites and pathways that need to be further investigated for their possible implications in this disease and for their therapeutic potentials. This feasibility study on oral signature in MS patients must be evaluated and reproduced using larger datasets.

## Materials and methods

### Patient samples

Participants were recruited at the MS Center (CRC-SEP Pays de La Loire) and samples were stored in a biocollection facility of Nantes University Hospital (ABM PFS13-003 “Collection sclérose en plaques”). All participants in the current study provided their informed consent for the analysis of their samples. Relapsing–remitting (RR) MS patients were recruited based on the revised McDonald criteria^43^ at the Nantes University Hospital and sampled during hospitalization. Patients with primary progressive multiple sclerosis (PPMS) and other neurological diseases or disorders (neuromyelitis optica spectrum disease (NMO-SD), clinically isolated syndrome not reaching McDonald criteria), or patients receiving antibody-based immunotherapy treatments or disease-modifying treatment (DMT) were excluded. Healthy volunteers (HVs) were recruited from among medical staff in the same hospital department. All participants had European ancestry. Patients who were obese, had a history of veganism or were using probiotics were also excluded. The exclusion criteria also included viral infection or the use of antibiotics drugs in the past three months. Overall, 34 saliva samples (21 HV, 14 RRMS) and 23 faecal samples (11 HV, 12 RRMS) were included in the microbiota analysis (Fig. 1). The groups were matched for age, sex, and smoking status. Five RRMS patients received intravenous corticosteroids (Solumedrol) at a dose of 1 g per day 1 to 3 days prior to saliva and faeces sampling. The MS patients included in the oral microbiota characterization had a mean disease duration of 3.17 years, a mean expanded disability status scale (EDSS) score of 1.9, and 11/14 of them were experiencing relapse at the time of sampling (Fig. 1A). Overall, the MS patients included for faecal microbiota analysis had a mean disease duration of 1.6 years and a mean EDSS of 1.2, and 11/12 of them were experiencing relapse at the time of sampling.

### 16S rRNA sequencing

DNA was extracted from 700 µL of saliva and 250 mg of stool using the QIAamp PowerFecal Pro DNA Kit from QIAGEN (Qiagen Inc., Venlo, The Netherlands). DNA spectrometry quality controls were used and all DNA samples had OD260:OD280 ratios greater than 1.8. 16S rRNA genes were amplified using the V4 553F and 805R primers via the classical two-step PCR approach, and paired-end sequencing of the PCR products was performed by a Miseq illumina system. Appropriate negative controls were included as sequencing blanks to assess bacterial contamination. Basic processing of the raw data was performed by the University of Illinois at Chicago Research Informatics Core (UICRIC, Chicago, IL, USA). Most of the quality control checks in the pipeline used were pass/fail, rather than comparison to a threshold. Read pair without merging, merging read without beginning or ending with the primers and chimera were excluded. Sequence trimming used cutadapt to remove ambiguous nucleotides, primer sequences, and trimmed was based on a quality threshold of p = 0.01. Reads that lacked either primer sequence, identified with a maximum mismatch of 10%, or were less than 225 bp were discarded.

### Pipeline analysis of the microbiota

The raw demultiplexed paired-end reads were merged using PEAR software^44^. First quality control and trimming were performed using Cutadapt^45^. Taxonomy amplicon sequencing variants were obtained from the QIIME2 DADA2 analysis pipeline using a naive Bayesian model trained on the SILVA 16S database^46,47^. Differentially abundant taxa at the genus level were evaluated using nonparametric Wilcoxon tests corrected for permutation-based false discovery rate (FDR) as well as linear discriminant analysis (LDA) using the LEfSe tool^48^, which was developed by the Huttenhower Laboratory (https://huttenhower.sph.harvard.edu). Diversity indices were evaluated based on the Shannon index and specific richness while β-diversity and overall dysbiosis status were evaluated through PERMANOVA^49^ applied to a Bray–Curtis dissimilarity matrix, and represented using principal coordinates analysis (PCoA).

Metagenomic gene content and pathway enrichment data were obtained using the PICRUSt2 pipeline^28^ based on inferred unobserved traits. Microbiota-derived metabolites were evaluated using custom in silico community flux balance analysis (FBA) based on the cobra python library^50^. Briefly, FBA is used to calculate reaction fluxes from genome-scale reconstruction models by minimization of an objective function. Outputs corresponding to boundary reactions provide insights into the metabolites that are consumed or produced by the considered organism or community. For each individual, the most prevalent taxa were selected and their metabolomic reconstitution models were retrieved from the Virtual Metabolic Human platform (https://www.vmh.life). All available reconstruction models were constructed for the genera representing more than 1% of the total genera in at least one individual. Models were weighted according to the relative abundance of the individual taxa in each individual and merged into a unique model representing a microbiome reconstitution. Output boundary reactions were considered to infer microbiome-derived metabolite content. The boundary reaction fluxes, corresponding to the produced and consumed metabolites were subsequently transformed into a bioavailability index consisting of the fold change between the MS and HV samples.

*t-SNE* dimensional reductions were performed on both taxonomy (at the genus level) and metabolic fluxes to observe the discrimination power of each of the two levels of resolution in the faecal and oral samples. Finally, we created a discrimination model based on logistic regression analysis of the microbiome-derived metabolites and tested it on an oral microbiome analysis of an independent set of individuals, performed at Kiel University (Kiel, Germany) using 16S rRNA^23^. Briefly, taxonomic tables were gathered for 38 HV and 12 MS patients free of any DMT. As above, metabolic reconstructions were performed using FBA and the boundary reaction fluxes were used to assess the reproducibility of our discrimination model. Similarly, we investigated the specificity of the oral microbiome-derived signature found in MS by comparing our data to data from rheumatoid arthritis (RA) patients, by collecting raw 16S rRNA reads from previously published data^51^ involving saliva samples from 22 HV and 28 RA patients. The raw 16S rRNA reads were processed as previously described and a community FBA model was established. The resulting fluxes were corrected for batch effects, merged with the reactional fluxes previously obtained for the MS patients and presented on a t-SNE dimensional reduction plot.

### Quantitative PCR

Quantitative PCR (qPCR) was performed to quantify spermidine synthase (speE, EC: 2.5.1.16) and nitrite reductase (nrfA, EC: 1.7.2.2) gene expression. The gene sequences expressed by different bacterial genera were aligned via Clustalo (https://www.ebi.ac.uk/Tools/msa/clustalo) and degenerate primer pairs were generated from the resulting consensus sequence using PrimerBlast (https://www.ncbi.nlm.nih.gov/tools/primer-blast/); we ensured the exclusion of human sequences to ensure high specificity. For each target gene, we used two endogenous controls, one universal 16S rRNA primer pair and one primer pairs specific for the 16S rRNA of a bacterial genus, which exhibited low variability between individuals (Table S3). For faecal speE, we used universal 16S rRNA primers 341S and 518R and primers specific for *Bacteroides*. For oral nrfA, we used the universal 16S rRNA primers 341S and 518R and primers specific for *Veillonella.* All primers were validated on a pool of all samples and showed high specificity for the targets and amplification efficacies between 90 and 110%. Quantitative PCR was performed on the same DNA as used for microbiome profiling: In total 11 RRMS and 18 HV samples were used for oral nrfA quantification while 11 HV and 12 RRMS samples were used for faecal speE quantification. PCR was carried out using QuantStudio 3 Real-time PCR system. For each target, relative levels were determined using the delta-delta CT method.

### Mass spectrometry

Salivary levels of deoxyadenosine were determined by liquid chromatography-tandem mass spectrometry (MS) on a Xevo^®^ Triple-Quadrupole mass spectrometer with an electrospray ionization interface equipped with an Acquity H-Class^®^ UPLC™ device (Waters Corporation, Milford, MA, USA). All solvents used were LC–MS grade and purchased from Biosolve (Valkenswaard, Netherlands), and standard compounds and internal standards (IS) were obtained from Sigma Aldrich (Saint-Quentin Fallavier, France). Individual deoxyadenosine stock solutions were prepared at 10 mmol/L in ultrapure water. A solution of labeled [^13^C_10_,^15^N_5_]-adenosine (25 µmol/L) was prepared in water containing 3 g/L bovine serum albumin (IS solution). The standard solutions and saliva samples (50 µL) were then extracted with 150 µL of acetonitrile and 50 µL of the IS solution. The samples were mixed and centrifuged at 10,000×*g* and 10 °C for 10 min to remove the precipitate proteins. The supernatants were dried under a gentle stream of nitrogen (45 °C) and dissolved in 100 µL of acetonitrile. The samples were injected onto an Acquity BEH-Amide column held at 60 °C, after which the compounds were separated. The target compounds were detected by mass spectrometer with an electrospray ionization operating in positive ion mode.

### Statistical analyses

Basic clinical variables were compared using the nonparametric Wilcoxon and McNemar tests. We investigated α-diversity (specific richness and Shannon index) and β-diversity (based on Bray–Curtis dissimilarity matrix) indices. Changes in α-diversity indices (Shannon index and specific diversity) were evaluated using Wilcoxon tests, and changes in β-diversity were tested through PERMANOVA using 1000 permutations and applied to the Bray–Curtis dissimilarity matrix. All diversity indices were obtained and processed using the Vegan R package v2.5-7. At the taxonomic and metabolite levels, fluctuations in relative abundances for each identified genus were tested using Wilcoxon tests corrected for permutation-based FDR. The discriminative power of taxa and metabolites was evaluated using t-SNE representations and logistic regression was performed using stepwise variable selection of the oral microbiota-derived metabolite fluxes to create the discrimination model. Gut and oral microbiota comparisons were made using Spearman correlation indices and the significance level was investigated using the Wilcoxon signed-rank test.

### Ethics approval and consent to participate

The relevant institutional review boards/ethics committees approved the protocol: Agence de Biomédecine (ABM PFS13-003 Collection sclérose en plaques). All donors provided written informed consent in compliance with the Declaration of Helsinki.

### Supplementary Information

Supplementary Figures.

Supplementary Table S1.

Supplementary Table S2.

Supplementary Table S3.

## Data Availability

The data set generated and analyzed for the current study is available in the European read archive repository (https://www.ebi.ac.uk/ena/browser/home) under the accession number PRJEB53481. Anonymized metadata will be available on reasonable demand to laureline.berthelot@inserm.fr.
