# Supplementary material for: Investigating the metabolite signature of an altered oral microbiota as a discriminant factor for multiple sclerosis: a pilot study
Source: Sci Rep. 2024 Apr 2;14:7786. doi: 10.1038/s41598-024-57949-4 (PMC10987558; doi:10.1038/s41598-024-57949-4)
Supplement: Supplementary file 1 — Supplementary Figures. [file 41598_2024_57949_MOESM1_ESM.docx]

**Supplementary figures:**


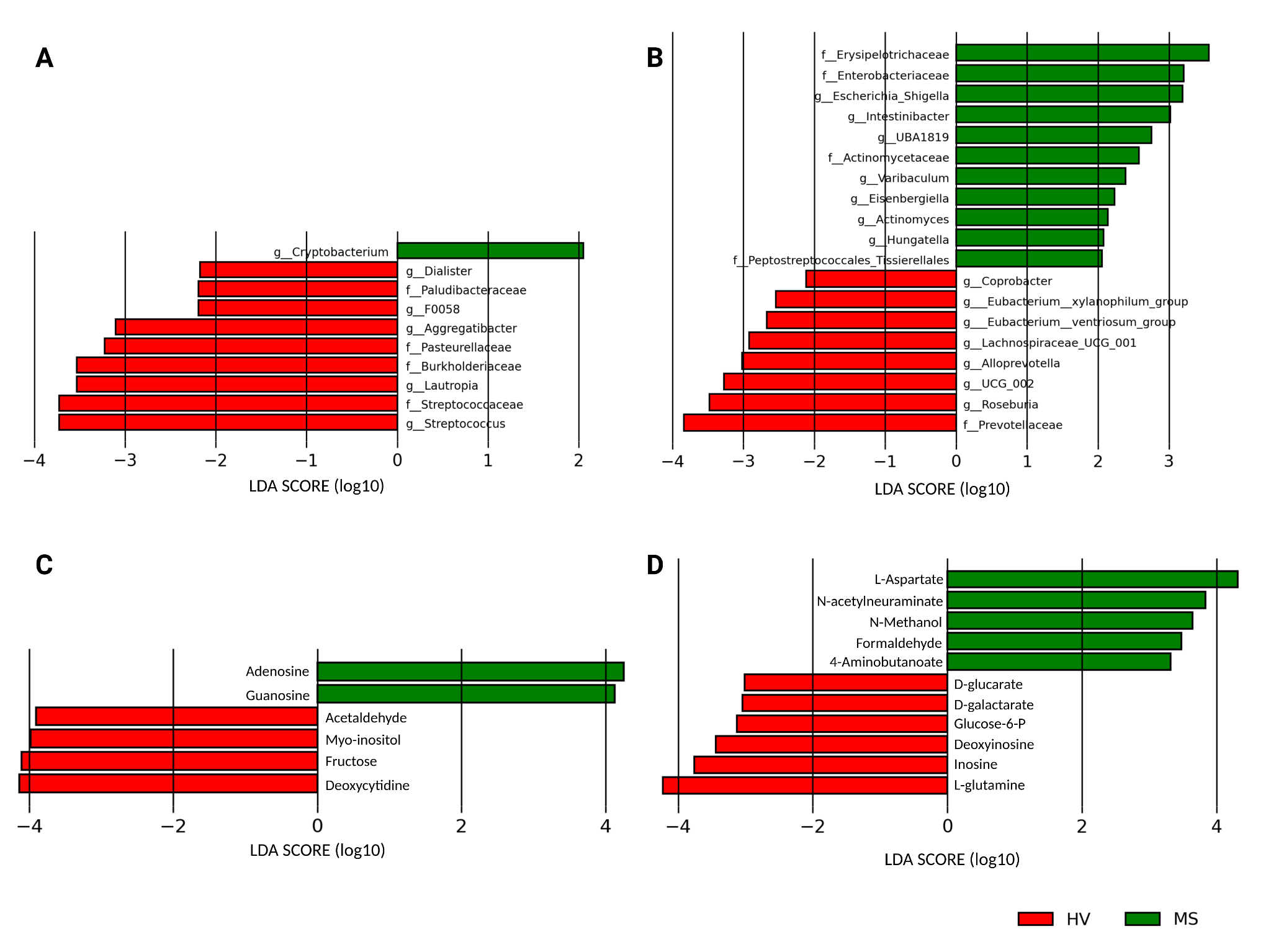


***Figure S1****: LDA analysis: calculation of the most discriminating variables between HV and MS patients for A: Oral microbiome taxonomy, B:  Fecal microbiome taxonomy, C: Oral microbiome-derived metabolites bioavailability, D: Fecal microbiome-derived metabolites bioavailability*

*
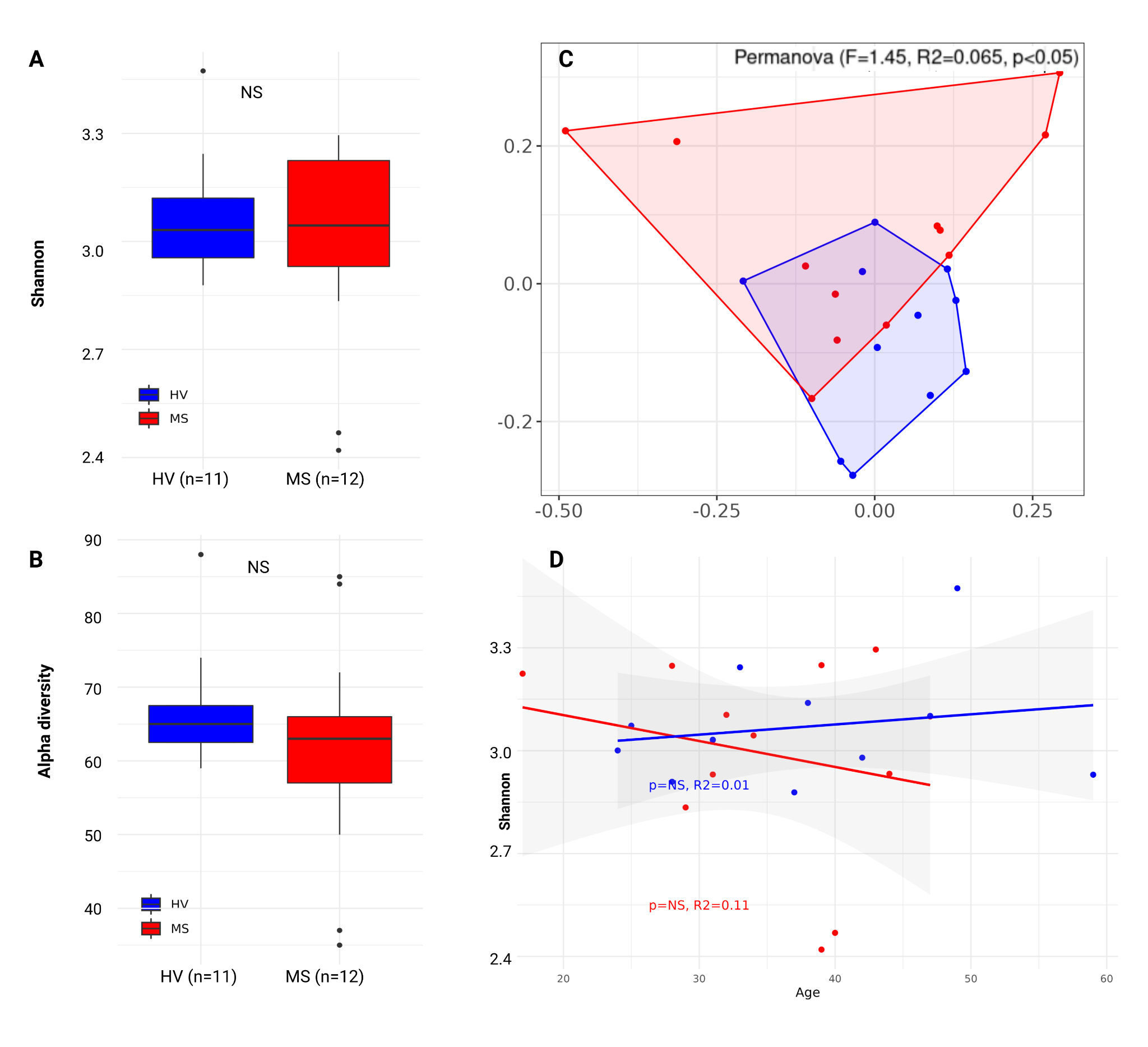
*

***Figure S2****: Fecal diversity indices: α diversity and Shannon indices are not different between MS and HV (A, B). Bars represent standard deviation (SD).* β*-diversity PERMANOVA analysis showed differences in the overall microbiome of MS patients compared to HV, confirming dysbiosis state in the gut of MS patients (C, p<0.05). Shannon index from feces microbiome is not associated with age (D).* β*-diversity PERMANOVA analysis.*

**
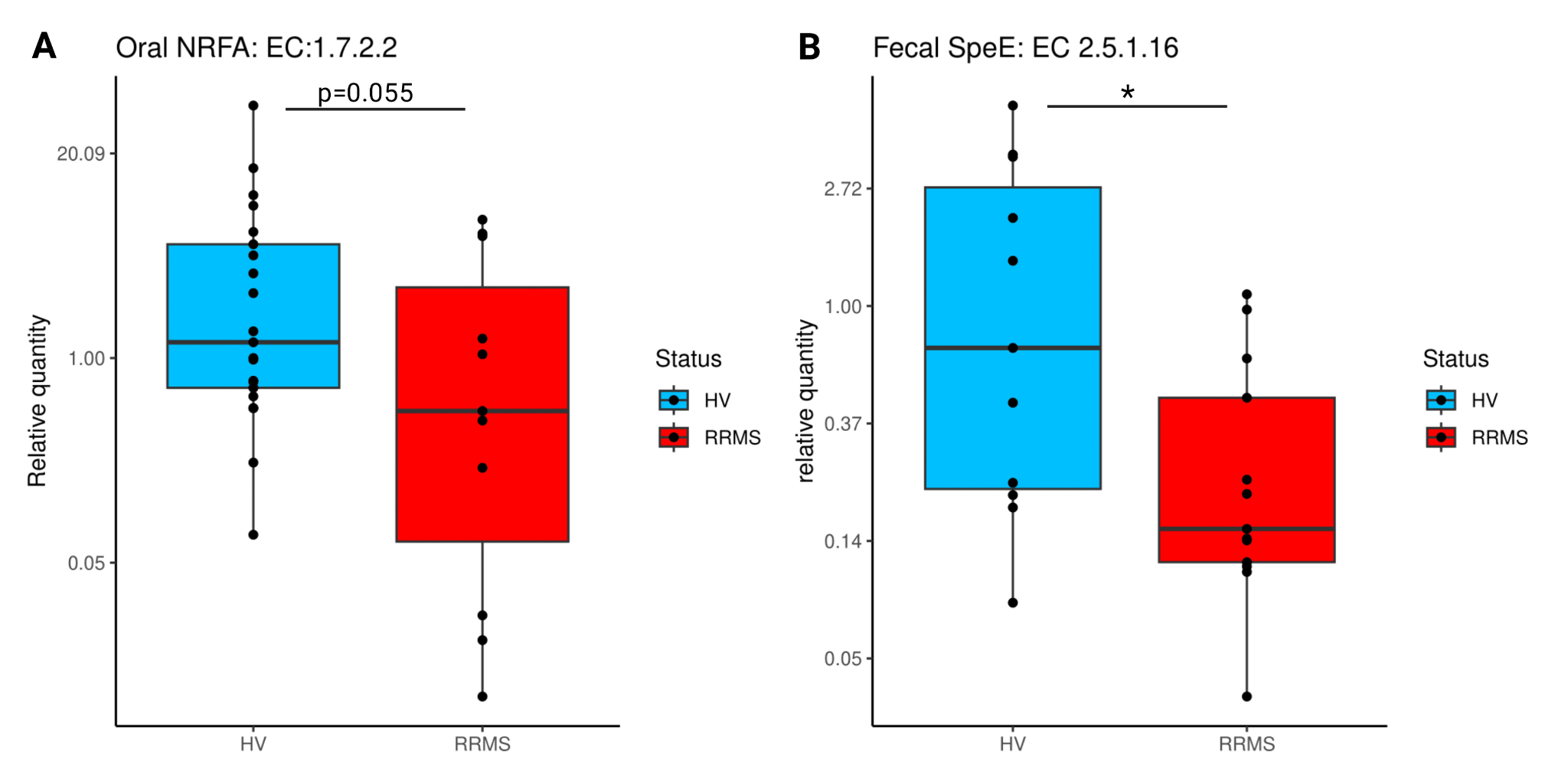
**

***Figure S3****: qPCR relative quantification of gene copy number of A: Oral nitrite reductase and B: Fecal Spermidine synthase, predicted to be altered in MS by Picrust analysis*


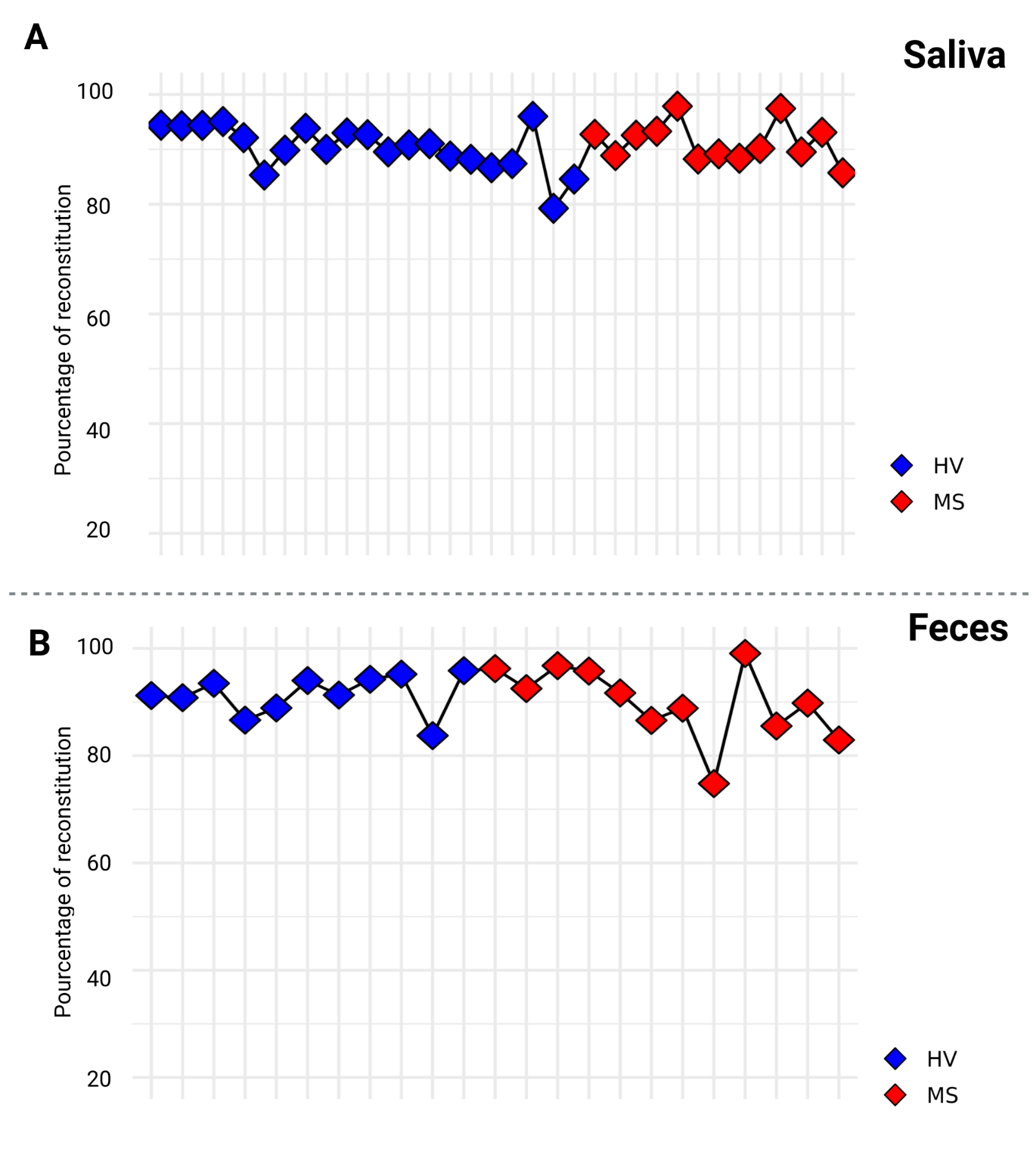


***Figure S4****: Levels of metabolic reconstruction for each individual. Bacterial metabolism models were merged together and pondered to reflect the abundance of each bacteria for a given individual.* ***A****: Oral microbial metabolism reconstruction,* ***B****: Fecal microbial metabolism reconstructions.*
