# Supplementary material for: Investigating the metabolite signature of an altered oral microbiota as a discriminant factor for multiple sclerosis: a pilot study
Source: Sci Rep. 2024 Apr 2;14:7786. doi: 10.1038/s41598-024-57949-4 (PMC10987558; doi:10.1038/s41598-024-57949-4)
Supplement: Supplementary file 4 — Supplementary Table S3. [file 41598_2024_57949_MOESM4_ESM.docx]

Table S3: Primer characteristics used for Quantitative PCR experiment

| **target** | **Sequence 5’ 3’** | **tm** | **prod size** | **start -end** | **Size primer** | **Direction** |
| --- | --- | --- | --- | --- | --- | --- |
| speE | GATCAGCCGGGTGTATCAGG | 59.97 | 71 | 662-681 | 20 | Forward |
| speE | TGGTTGCAAAACCGAACAGC | 60.18 | 71 | 732-713 | 20 | Reverse |
| nrfA | AAAGGTCCGGATGTTCCACG | 60.32 | 118 | 456-475 | 20 | Forward |
| nrfA | TGACAGTCAGCACAACCGAT | 59.61 | 118 | 573-554 | 20 | Reverse |
| 16s_341S | TTACCGCGGCTGCTGG | 61 | 177 | 341-518 | 16 | Forward |
| 16s_518R | CCTACGGGNGGCWGCAG | 61 | 177 | 341-518 | 17 | Reverse |
| 16s_bacteroides | CGGTAAAAGATGGGGATGCG | 59.06 | 209 | 223-242 | 20 | Forward |
| 16s_bacteroides | ACCCATAGGGCAGTCATCCT | 60.03 | 209 | 431-412 | 20 | Reverse |
| 16s_ veillonella | AGTCATTCACACCCGAAGCC | 60.32 | 78 | 1439-1458 | 20 | Forward |
| 16s_veillonella | ACTTCACCCCAATCATCGCC | 60.39 | 78 | 1516-1497 | 20 | Reverse |
